# Preclinical Assessment of Viral Vectored and Protein Vaccines Targeting the Duffy-Binding Protein Region II of *Plasmodium Vivax*

**DOI:** 10.3389/fimmu.2015.00348

**Published:** 2015-07-08

**Authors:** Simone C. de Cassan, A. Rushdi Shakri, David Llewellyn, Sean C. Elias, Jee Sun Cho, Anna L. Goodman, Jing Jin, Alexander D. Douglas, Rossarin Suwanarusk, François H. Nosten, Laurent Rénia, Bruce Russell, Chetan E. Chitnis, Simon J. Draper

**Affiliations:** ^1^The Jenner Institute, University of Oxford, Oxford, UK; ^2^International Center for Genetic Engineering and Biotechnology, New Delhi, India; ^3^Department of Microbiology, Yong Loo Lin School of Medicine, National University Health System, National University of Singapore, Singapore, Singapore; ^4^Singapore Immunology Network, Agency for Science, Technology and Research (A*STAR), Singapore, Singapore; ^5^Shoklo Malaria Research Unit (SMRU), Mahidol-Oxford Tropical Medicine Research Unit, Faculty of Tropical Medicine, Mahidol University, Mae Sot, Thailand

**Keywords:** malaria, vaccine, *Plasmodium vivax*, blood-stage, adenovirus, poxvirus, MVA, Duffy-binding protein

## Abstract

Malaria vaccine development has largely focused on *Plasmodium falciparum*; however, a reawakening to the importance of *Plasmodium vivax* has spurred efforts to develop vaccines against this difficult to treat and at times severe form of relapsing malaria, which constitutes a significant proportion of human malaria cases worldwide. The almost complete dependence of *P. vivax* red blood cell invasion on the interaction of the *P. vivax* Duffy-binding protein region II (PvDBP_RII) with the human Duffy antigen receptor for chemokines (DARC) makes this antigen an attractive vaccine candidate against blood-stage *P. vivax*. Here, we generated both preclinical and clinically compatible adenoviral and poxviral vectored vaccine candidates expressing the Salvador I allele of PvDBP_RII – including human adenovirus serotype 5 (HAdV5), chimpanzee adenovirus serotype 63 (ChAd63), and modified vaccinia virus Ankara (MVA) vectors. We report on the antibody and T cell immunogenicity of these vaccines in mice or rabbits, either used alone in a viral vectored prime-boost regime or in “mixed-modality” adenovirus prime – protein-in-­adjuvant boost regimes (using a recombinant PvDBP_RII protein antigen formulated in Montanide^®^ISA720 or Abisco^®^100 adjuvants). Antibodies induced by these regimes were found to bind to native parasite antigen from *P. vivax* infected Thai patients and were capable of inhibiting the binding of PvDBP_RII to its receptor DARC using an *in vitro* binding inhibition assay. In recent years, recombinant ChAd63 and MVA vectors have been quickly translated into human clinical trials for numerous antigens from *P. falciparum* as well as a growing number of other pathogens. The vectors reported here are immunogenic in small animals, elicit antibodies against PvDBP_RII, and have recently entered clinical trials, which will provide the first assessment of the safety and immunogenicity of the PvDBP_RII antigen in humans.

## Introduction

Malaria is caused by protozoan parasites of the genus *Plasmodium*, with five species known to infect humans. Despite extensive efforts with existing control measures, the levels of morbidity and mortality associated with this disease remain sobering. Historically, vaccine development efforts have almost exclusively focused on *Plasmodium falciparum* – the major causative agent of malarial disease in sub-Saharan Africa (1). A second species of parasite, *Plasmodium vivax*, is the most widespread geographically and thus constitutes a significant proportion of human malaria cases worldwide. Although viewed for many decades as a relatively inconsequential parasite (2), revised estimates suggest 2.5 billion people are living at risk of *P. vivax* infection in the Americas, as well as Central and South-East Asia (3). Recent data also demonstrate that the infection brings a significant burden of morbidity and associated mortality, which has been largely under-appreciated in the past (4). Consequently, the recently revised Malaria Vaccine Technology Roadmap to 2030 (5) now recognizes the importance of *P. vivax* malaria and calls for a vaccine to achieve 75% efficacy over 2 years – equally weighted with *P. falciparum* in an era of renewed political will to control and eradicate this devastating disease.

Different stages of the malaria parasite’s life-cycle can be targeted by subunit immunization. In the past, a small handful of pre-erythrocytic and sexual-stage vaccine candidates for *P. vivax*, based on the PvCSP and Pvs25 antigens respectively, have entered early phase clinical testing as recombinant protein or long synthetic peptide in adjuvant formulations (6–9); none of these candidate formulations, however, remain in active clinical trials. Vaccines targeting the asexual blood-stage infection remain an alternative approach, aiming to control and clear parasitemia in order to prevent disease and death as well as continued transmission. A number of blood-stage malaria vaccine candidates, mainly focusing on merozoite ligands involved in erythrocyte invasion, are under development for *P. falciparum* (10) but, as yet, no clinical trials of equivalent *P. vivax* blood-stage candidate vaccines have been reported (11).

Merozoite invasion of erythrocytes is a complex, multi-step process involving many receptor–ligand interactions between the parasite and the surface of the host’s red blood cell (RBC) (12). Invasion of RBCs by *P. vixax* is restricted to CD71^+^ reticulocytes (13) and commonly uses the interaction of the *P. vivax* Duffy-binding protein (PvDBP) with the human Duffy antigen receptor for chemokines (DARC/Fy) (14). Notably, Duffy-negative individuals appear to be protected from blood-stage *P. vivax* infection, an observation first reported by Miller et al. in 1976 (15), confirmed by controlled human infection studies (16), and associated geographically with sub-Saharan Africa where *P. vivax* is largely absent (17). Of note, there have been reports of *P*. *vivax* isolates that can invade Duffy-negative cells (18), with recent sequencing data identifying a gene encoding a PvDBP paralog (19). These data suggest that increased expression levels or gene copy number may enable invasion into Duffy-negative cells, and further highlight the importance of the PvDBP antigen in *P*. *vivax* infection.

The micronemal parasite ligands (DBPs or erythrocyte-binding ligands/antigens, EBL/EBA) are a family of antigens that are functionally conserved across *Plasmodium* species. All parasites have at least one EBL, and in many cases these lead to redundancy, as observed in *P. falciparum* (20). In the case of *P. vivax*, the PvDBP gene and its paralog are known to exist (19), and genetic knockout of the orthologous simian malaria *P. knowlesi* DBPα gene prevents invasion of Duffy-positive erythrocytes *in vitro* (21). The receptor-binding domain of PvDBP lies within the conserved, extracellular, cysteine-rich region known as region II (PvDBP_RII) (22). Antibodies can be induced against this antigen in mice and rhesus macaques using recombinant PvDBP_RII protein (rDBP)-in-adjuvant vaccines (23, 24), and those raised against the *P. knowlesi* DBPα ortholog can block RBC invasion by this parasite *in vitro* (25). Furthermore, naturally acquired high-titer binding inhibitory antibodies against PvDBP_RII have been shown to be associated with reduced risk of *P. vivax* infection in children residing in an endemic area, as well as lower *P. vivax* parasite densities following infection (26). Thus, to date, the PvDBP_RII adhesin remains the most promising subunit vaccine target against *P. vivax* merozoites; however, this antigen has never been progressed to clinical trials and, consequently, no data have existed on the ability of vaccines to induce effective immune responses in humans.

Traditionally, recombinant protein vaccines have been developed when seeking to induce antibodies by vaccination. Development of such vaccines requires production of the antigen in a heterologous expression system followed by formulation in a suitable human-compatible adjuvant (27). An alternative approach, developed in recent years, has used recombinant viral vectored vaccines to deliver proteins of interest with the key aim of inducing antibodies in conjunction with T cell responses. A strategy demonstrating the highest degree of success to date has utilized a recombinant replication-deficient adenovirus to prime an immune response, followed by a booster vaccination (typically 8 weeks later) with an attenuated poxvirus recombinant for the same antigen (28). This approach has been shown to be reliably immunogenic for high-titer antibody induction against a variety of difficult-to-express malaria antigens in mice, rabbits, and non-human primates (NHP) (29–32). It has also been shown to be safe and immunogenic for the delivery of the *P. falciparum* blood-stage antigens merozoite surface protein 1 (PfMSP1) and apical membrane antigen 1 (PfAMA1) in a series of Phase I/IIa clinical trials in healthy adult UK volunteers (33), and the same viral vectored vaccine technologies are currently entering Phase II/III clinical testing in West Africa for Ebola (34).

An extension of this approach has seen the development of “mixed modality” adenoviral prime – protein-in-adjuvant boost (AP) regimes, whereby the two leading subunit vaccine delivery platforms are combined, often leading to improved immune responses in mice in comparison to the use of either strategy alone (35, 36). In agreement with these murine data, NHP studies of similar regimes, for candidate malaria and HIV-1 vaccines, have also shown particular promise (30, 37, 38). Here, we generated both preclinical and clinically compatible adenoviral and poxviral vectored vaccine candidates expressing PvDBP_RII. We report on the humoral and cellular immunogenicity of these vaccines in mice or rabbits, either used alone in a viral vectored prime-boost regime or in AP regimes using a recombinant protein PvDBP_RII antigen administered in adjuvant. Antibodies induced by these regimes were found to bind to native parasite antigen from *P. vivax* infected patients and were capable of inhibiting the binding of PvDBP_RII to its receptor DARC *in vitro*.

## Materials and Methods

### Vaccines and protein

Viral vectored vaccines expressing PvDBP_RII were generated according to previously described methods. Recombinant viruses each express the 984 bp coding sequence of region II of the Duffy-binding protein from the Salvador I (*Sal*I) strain of *P. vivax*, amino acids (αα) 194–521 (GenBank Accession #DQ156512). The PvDBP_RII construct was codon optimized for human expression and synthesized by GeneArt (Regensburg, Germany). The PvDBP_RII sequence was cloned in frame at the N-terminus to the human tissue plasminogen activator (tPA) leader sequence (αα 1–32, GenBank Accession # K03021), which in turn was preceded by a Kozak sequence (29). This transgene cassette was inserted using Gateway^®^ site specific recombination technology (Invitrogen) into the E1 site of vectors encoding the genomes of E1/E3-deleted HAdV5 (29) and ChAd63 (39), under the control of the 1.9 kbp CMV promoter (40). The entire E4 locus of the ChAd63 vector is also replaced with the *E4Orf6* gene from HAdV5 as previously described (39). The recombinant adenoviral vaccines were generated and cultured in HEK293 cells, purified by CsCl centrifugation, and titered by UV spectrophotometry to give units of viral particles (vp/mL) (41) or by anti-hexon immunostaining of infected T-REx-293 cells to give infectious units (ifu/mL) (31). In the case of modified vaccinia virus Ankara (MVA), the transgene cassette was recombined into the thymidine kinase (TK) locus of MVA with expression driven by the vaccinia P7.5 early/late promoter. Recombinant MVA viruses were generated as previously described – either with an additional GFP marker gene (29) or without any additional marker (35). Both MVA vaccines were grown in chicken embryo fibroblasts (CEFs), purified by centrifugation through a sucrose cushion, and titered by plaque assay to give plaque-forming units (pfu/mL) (41). Control vectors expressing ovalbumin (OVA) have been described previously (36).

Unless otherwise stated, all studies used rDBP produced in *Escherichia coli* and purified as previously described (42). Endotoxin levels were measured using the Limulus amebocyte lysate (LAL) gel-clot assay according to the manufacturer’s instructions (Salesworth). The endotoxin content was <22 EU per 25 μg protein.

For the rabbit serum ELISAs, rDBP was produced in suspension HEK293E cells. The construct included from N- to C-terminus: the human tPA leader sequence and PvDBP_RII (as for the viral vector vaccines), followed by an AviTag biotin acceptor peptide (amino acids GLNDIFEAQKIEWHE) followed by a hexa-histidine (His6) tag. Six liters of suspension HEK293E cells were transiently transfected as previously described (32), and culture supernatants were harvested after 4 days when cell viability fell below 95%. This was then concentrated 20-fold and subsequently buffer exchanged into PBS using a Pellicon 3 tangential flow filtration (TFF) system (Millipore, Herts, UK). Purification consisted of a cobalt-based immobilized metal affinity chromatography (IMAC) – Hitrap TALON crude (GE Healthcare, Bucks, UK) and a size exclusion chromatography (SEC) – SepFast GF-HS-L 16/1000 (Biotoolomics, Durham, UK). The purified protein was quantified by Nanodrop (Thermo Fisher Scientific, Leics, UK) and stored at −80°C until further use.

### Vaccine adjuvants

Adjuvants used in this study were dosed and prepared in low phosphate PBS (<5mM) (Gibco-Invitrogen, UK) as previously described (36). In brief, Abisco^®^100 (Isconova, Sweden) (12 μg/dose) was gently mixed with antigen in PBS; and Montanide^®^ISA720 (Seppic, France) was emulsified using a T10 ULTRA-TURRAX^®^ (IKA^®^) homogenizer under sterile conditions at 25,000 rpm for 6 min, keeping the sample on ice in a ratio of 3:7 (antigen:adjuvant). All vaccines were kept on ice until administration. Abisco^®^100 is an ISCOMatrix containing phosphatidyl choline phospholipid, cholesterol, and saponins purified from the tree *Quillaja saponaria* Molina – a mixture of Matrix A and Matrix C (containing the QS7 and QS21 fractions respectively) purified from the Quil-A extract. Montanide^®^ISA720, an oil-in-water emulsion, is produced with squalene oil and a refined surfactant based on mannide oleate.

### Animals and immunizations

All procedures on mice were performed in accordance with the terms of the UK Animals (Scientific Procedures) Act Project License and were approved by the University of Oxford Animal Welfare and Ethical Review Body. Six to eight-week-old female BALB/c (H-2^d^) mice were purchased from Harlan Laboratories (Oxfordshire, UK) or bred at the Wellcome Trust Centre for Human Genetics (Oxford, UK). Mice were anesthetized before immunization with Isoflo (Abbot Animal Health, UK), and all immunizations were administered intramuscularly (i.m.) with vaccine divided equally into each *musculus tibialis*. Immunization doses and regimes are explained in the text and figure legends. Serum was harvested at stated time-points from tail vein bleeds or by exsanguination under terminal anesthesia at the final harvest time-point.

Rabbit work was conducted by Biogenes (Germany). Female ZiKa rabbits (*n* = 4) were immunized i.m. with 4 × 10^8^ ifu HAdV5-PvDBP_RII on day 0 and 5 × 10^7^ pfu MVA-PvDBP_RII (ML) on day 56. Two control rabbits received the same doses and regime of viral vectors encoding OVA (36). Serum was harvested before immunization (day 0) and 28, 56, and 70 days post adenovirus administration.

### Enzyme linked immunosorbent assay

Unless stated otherwise, mouse IgG ELISAs were carried out using a standardized ELISA according to previously described methodology (43, 44) and using a reference sample generated from high-titer sera pooled from PvDBP_RII vaccinated mice. A 1:9000 dilution of the reference sample gave an OD_405_ = 1.0, and thus this reference serum was taken to be 9000 arbitrary units (AU). Test samples were diluted appropriately so that their optical density 405 nm (OD_405_) could be read off the linear part of the reference curve. Alternatively, and where stated in the text, endpoint ELISAs were performed as described previously (29). In brief, Nunc-Immuno Maxisorp plates (Thermo Scientific, UK) were coated overnight at room temperature (RT) with PvDBP_RII protein. Plates were washed with PBS/0.05% Tween 20 (PBS/T) and blocked for 1 h with 10% skimmed milk powder in PBS/T. Serum samples were diluted in PBS/T and added in duplicate, with threefold dilutions down the plate, before incubation for 2 h at RT. After washing, goat anti-mouse total IgG conjugated to alkaline phosphatase (Sigma Aldrich, UK) was added for 1 h at RT, followed by a final wash, and then addition of 1 mg/mL *p-*nitrophenylphosphate in diethanolamine buffer (Pierce, UK) as developing substrate. OD_405_ was read using a Model 550 Microplate Reader (Bio-Rad, UK). Serum antibody endpoint titers were taken as the *x*-axis intercept of the dilution curve at an absorbance value 3 SDs greater than the OD_405_ for naïve mouse serum. A standard positive serum sample and naïve serum sample were included as controls for each assay. The same methodology was used for rabbit endpoint ELISAs except that PvDBP_RII protein was produced in HEK293E cells and an anti-rabbit IgG secondary was used as described previously (45).

### Isotype ELISA

To measure antigen-specific IgG1 and IgG2a responses, plates were coated as before but at a concentration of 1 μg/mL protein. After blocking, serum diluted 1:1000 in PBS/T was added in duplicate to the plate for 2 h at RT. Plates were then washed and either biotin anti-mouse IgG1 or IgG2a (BD biosciences) at a 1:5000 dilution were added to the test plates for 1 h at RT. Following washing and a 30 min incubation with Extravidin Alkaline Phosphatase (Sigma Aldrich, UK), plates were developed as for total IgG ELISA. A standard positive serum sample and naïve mouse serum sample were included as controls.

### Isolation of splenocytes

Splenocytes were prepared fresh from mouse spleens as previously described (46). Where stated in the text, samples were also frozen down in 1 mL aliquots fetal calf serum (FCS) with 10% DMSO and stored at −80°C. Prior to use, frozen samples were thawed and immediately added to pre-warmed media [minimal essential media (MEM) α-modification containing 4 mM l-glutamine, 100 U/mL penicillin, 100 μg/mL streptomycin, 50 μM β-mercaptoethanol, and 10% heat-inactivated FCS]. Cells were then washed twice with media and allowed to rest at 37°C, 5% CO_2_ in a humidified incubator for 1 h before use.

### *Ex vivo* IFN-γ spleen ELISpot

IFN-γ ELISpots were performed using splenocytes as previously described (36). In brief, spleen cells were re-suspended at 1 × 10^7^ cells/mL in complete medium and plated at 50 μL cells per well. Fifty microliters complete medium alone was added to control wells, and 50 μL re-stimulation in complete medium was added to duplicate test wells as follows: rDBP at a final concentration 5 μg/mL; or a pool of 32 20mer peptides (NEO Peptide, USA) overlapping by 10 amino acids (except the final peptide, number 32, was an 18mer) corresponding to the whole of the PvDBP_RII coding sequence at a final concentration 5 μg/mL each peptide; or individual peptides used at 5 μg/mL final concentration. Results are expressed as spot forming units (SFU) per million splenocytes. Background responses in media-only wells were subtracted from those measured in re-stimulated wells.

### Intracellular cytokine staining

Mouse splenocytes were restimulated for 5 h at 37°C in the presence of GolgiPlug (BD Biosciences) and peptides [single or overlapping peptides (OLP) pool] specific for PvDBP_RII at a final concentration of 1 μg/mL per peptide (or no peptide unstimulated control), before storage at 4°C overnight. Cells were then incubated for 15 min in the presence of anti-mouse CD16/CD32 (Fc-block) before surface staining for 30 min, with anti-mouse CD8α-PerCP-Cy5.5 (clone 53-6.7) and CD4-Pacific Blue (clone GK1.5) and LIVE/DEAD Fixable Aqua Dead Cell Stain Kit for 405 nm excitation (Invitrogen). Permeabilization was performed using Cytofix/Cytoperm solution (BD Biosciences) according to the manufacturer’s instructions. Cells were stained intracellularly for 30 min with anti-mouse IFN-γ-Alexa Fluor 647 (clone XMG1.2). All incubations were performed at 4°C with staining antibodies from eBioscience. Cells were resuspended in 1% formalin solution and analyzed using a LSRII flow cytometer (BD Biosciences). Data were analyzed using FlowJo version 9.7.5. Background responses from the unstimulated control cells were typically 0.05%.

### Binding inhibition assay

A binding inhibition assay was carried out to study the ability of antibodies raised in mice against PvDBP_RII to inhibit the binding of this protein to its receptor DARC, as previously described (42, 47). Sera diluted as indicated were pre-incubated with 0.025 μg/mL of recombinant PvDBP_RII (*Sal*I strain sequence) for 1 h at RT before adding to ELISA plates pre-coated with recombinant DARC (nDARC-Fc; N-terminal extracellular 60 amino acids of DARC fused to Fc region of human IgG) protein at 1 μg/mL. Bound PvDBP_RII was detected with rabbit polyclonal anti-PvDBP_RII sera (1:4000 dilution), followed by goat anti-rabbit IgG HRP-conjugated antibodies (1:6000 dilution). A standard curve generated using binding of PvDBP_RII (0.002–0.025 μg/mL) to DARC was used to convert OD values into amount of protein bound in each well. Percent inhibition was calculated as 100% minus % protein bound. A positive control serum (raised in mice against PvDBP_RII using Freund’s adjuvant) and a negative control serum (from naïve mice) were included in each assay. The negative control sample had a median of 4% binding inhibition across all replicates tested across the different dilutions.

### Indirect immunofluorescence assay

*Plasmodium vivax*-infected blood was collected from malaria patients attending the clinics of the Shoklo Malaria Research Unit (SMRU), Mae Sot region northwest of Thailand, after written informed consent (OXTREC 027-025; University of Oxford, Centre for Clinical Vaccinology and Tropical Medicine, Oxford, UK). The *P. vivax*-infected erythrocytes were cultured to the late schizont stage prior to being concentrated and washed as previously described (48), before being smeared onto glass slides, air dried, and fixed with cold acetone for 20 min. For the immunofluorescence assays (IFAs), the thin-smear preparations were blocked with 1% Casein, 10% goat serum in PBS for 1 h at RT. Mouse sera (diluted 1:50) or rabbit sera (diluted 1:100) were applied to the slides and incubated for 1.5 h at 37.5°C or 1 h at RT in a humidified incubator, respectively. The slides were then washed three times with PBS before the addition of anti-mouse or anti-rabbit IgG conjugated to Alexa Fluor 488 (Molecular Probes) diluted 1:100 (mouse) or 1:1000 (rabbit) with 1% Casein, 10% goat serum in PBS for 1 h at RT, and mounted with Vectashield (Vectorlabs) with DAPI (4′,6-diamidino-2-phenylindole) (Invitrogen). Binding was visualized using a Nikon TS 100 epifluorescence microscope.

### Statistical analysis

All statistical analysis was carried out using Prism version 5.04 (Graphpad, USA). For non-parametric data, a Kruskal–Wallis test with Dunn’s multiple comparison post-test was used to compare more than two groups, and a Mann–Whitney *U*-test was used to compare two groups. A two-way ANOVA with Bonferroni’s multiple comparison post-test was used to explore the effect of two variables. *P* < 0.05 was considered significant.

## Results

### Antibody responses induced by adenovirus-MVA PvDBP_RII vaccines

Viral vectored vaccines, when used in a heterologous prime-boost regime, have previously been shown in various preclinical animal models to induce functional antigen-specific antibody responses targeting *P. falciparum* antigens, such as PfMSP1, PfAMA1, PfRH5, and Pfs25 (30–32, 45, 49, 50). Here, we initially generated recombinant viral vectors for use in preclinical proof-of-concept studies: a human adenovirus serotype 5 (HAdV5) vector expressing PvDBP_RII, and a MVA expressing the same PvDBP_RII antigen, as well as a selectable marker (green fluorescent protein, GFP) under the control of separate promoters.

**Figure 1:**
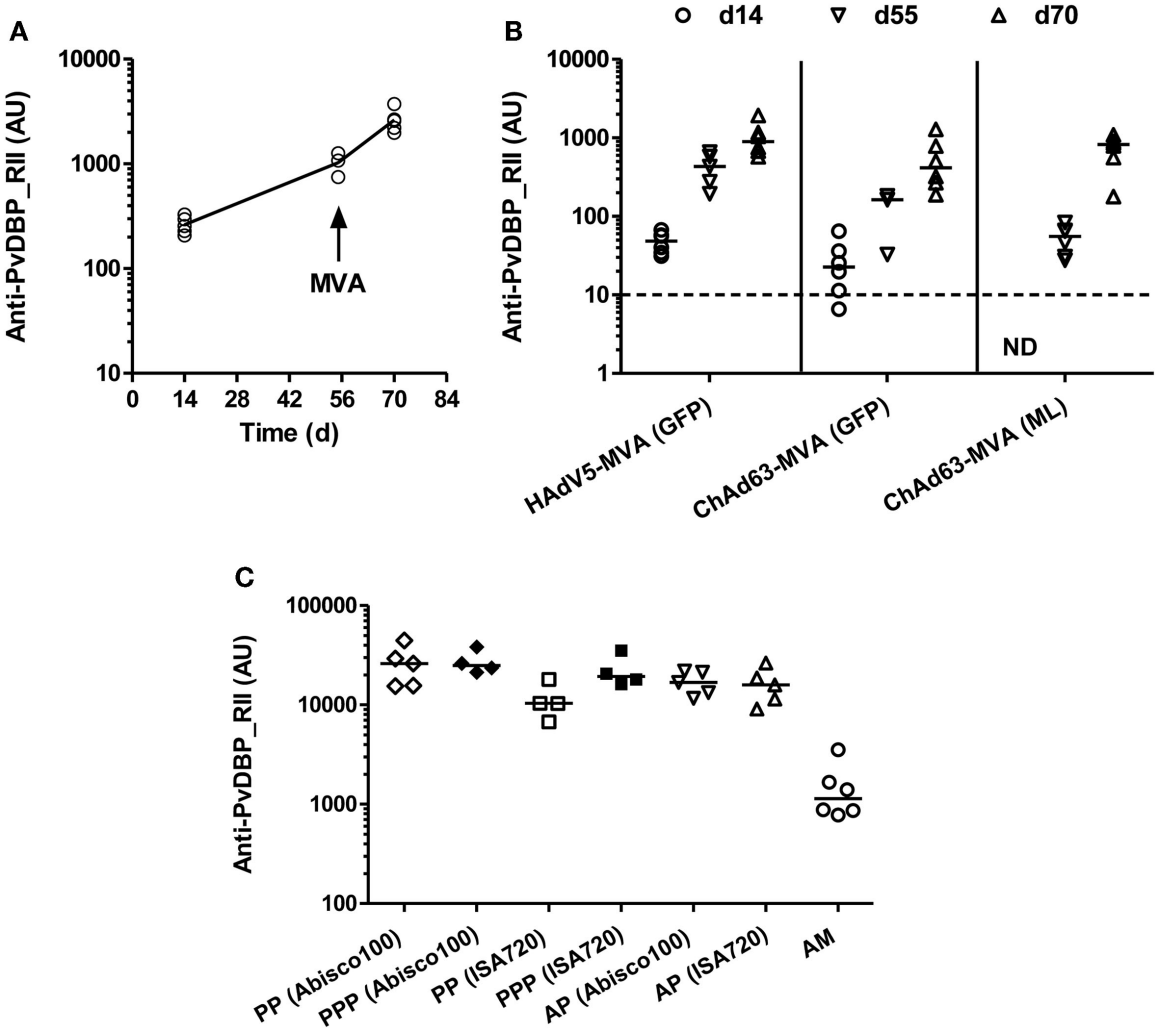
**Humoral responses induced by viral vectored and protein vaccines targeting PvDBP_RII**. **(A)** BALB/c mice (*n* = 5) were immunized with 10^10^ vp of a recombinant HAdV5 expressing PvDBP_RII, and boosted 8 weeks later with 10^7^ pfu of a recombinant MVA expressing PvDBP_RII and the selectable marker GFP. **(B)** BALB/c mice (*n* = 6/group) were immunized with 1.5 × 10^8^ ifu of either HAdV5 or ChAd63 expressing PvDBP_RII, and 8 weeks later, mice were boosted with 10^7^ pfu MVA-PvDBP_RII either expressing the selectable marker GFP or no marker (ML). **(C)** BALB/c mice (*n* = 4–6/group) were immunized with the regime outlined in **(A)** (AM), or immunized three times i.m. 3 weeks apart with 10 μg of PvDBP_RII protein formulated with the specified adjuvant (PPP), or primed i.m. with 10^10^ vp of HAdV5-PvDBP_RII and boosted 8 weeks later i.m. with 10 μg of PvDBP_RII protein formulated with adjuvant (AP). In all panels, serum IgG titers were measured in arbitrary units (AU) against PvDBP_RII protein by ELISA 2 weeks after each immunization (day 14 and 70) and before the boost (day 55) in **(A,B)**, and 2 weeks after the second immunization (PP) or final immunization (PPP, AP, AM) in **(C)**. In **(C)**, responses following two immunizations are shown with open symbols (PP, AP, AM) and after three immunizations with closed symbols (PPP). ND, not done. Median and individual data points are shown. The dotted line indicates the threshold for responses above background in **(B)** determined using serum taken from the mice prior to any immunization. The same cut-off would apply in **(A)** but is not indicated.

BALB/c mice were immunized with 10^10^ viral particles (vp) HAdV5-PvDBP_RII and boosted 8 weeks later with 10^7^ plaque forming units (pfu) MVA-PvDBP_RII (GFP). Total IgG titers were assayed against rDBP by ELISA in serum 2 weeks after each immunization and before the boost. Antigen-specific antibody responses were detected 14 days post-HAdV5 immunization (Figure 1A). These responses continued to increase by day 55 and were boosted by MVA vaccine administration on day 56. These responses confirmed the ability of viral vectored vaccines to induce anti-PvDBP_RII IgG.

Owing to high levels of pre-existing immunity to HAdV5 within the human population, other adenoviral vectors have been sought for clinical vaccine development (51). Simian adenoviruses, against which there is typically minimal neutralization by human sera, have emerged as a leading alternative (28, 52). In particular, the chimpanzee adenovirus serotype 63 (ChAd63) has been found to induce strong cellular and humoral immune responses, comparable to HAdV5 in animal models, and has also been shown to be safe and highly immunogenic in clinical trials (44, 53, 54). Having demonstrated the humoral immunogenicity of the preclinical HAdV5 and MVA vectors, we next sought to do the same with clinically compatible vectors and undertook a comparative immunogenicity study. We thus generated a ChAd63 vector expressing PvDBP_RII, as well as a recombinant MVA-PvDBP_RII vector lacking the GFP marker gene (“markerless”, ML) before undertaking a comparative immunogenicity assessment. Adenoviral vectors may be titered by either assessing vp or infectious units (ifu). Unlike vp counts, the ifu titer only measures virus capable of infecting cells, and therefore dosing based on ifu provides the most appropriate means for comparing adenoviral vector immunogenicity (39).

BALB/c mice were thus immunized with 1.5 × 10^8^ ifu HAdV5 or ChAd63 encoding PvDBP_RII, and 8 weeks later were boosted with 10^7 ^pfu MVA-PvDBP_RII (GFP) or MVA-PvDBP_RII (ML). As before, total IgG titers were assayed in serum 2 weeks after each immunization and before the boost by ELISA against PvDBP_RII protein. Following immunization with ChAd63-PvDBP_RII, the IgG responses followed the same antibody kinetic as observed with HAdV5. Similarly, there was no significant difference between total IgG titers 14 days after the priming immunization with HAdV5 or ChAd63 (*P* = 0.09, Mann–Whitney test, *n* = 6 per group), although there was a trend toward lower antibody titers with ChAd63 (Figure 1B). By day 55, this difference had become significant when IgG titers were compared between mice receiving HAdV5 versus ChAd63 (*P* = 0.0002, Mann–Whitney test, *n* = 6 versus 9); however, after the MVA boost immunization, there were no significant differences observed in the IgG titers at day 70 following the three different regimes tested (*P* = 0.11, Kruskal–Wallis test with Dunn’s multiple comparison post-test). These data showed the immunogenicity of the ChAd63 vector as well as the comparable immunogenicity of the markerless and GFP-expressing MVAs.

### Antibody responses induced by adenovirus-protein PvDBP_RII vaccines

We next assessed the ability of a “mixed modality” immunization approach (28) to maximize antibody induction by subunit vaccination. Adenovirus-protein/adjuvant (AP) and protein/adjuvant-only (PPP) regimes have previously been shown to induce higher titer antibody responses than the adenovirus-MVA regime (AM) in mice (35, 36), rhesus macaques (30), and humans (55). BALB/c mice were thus immunized with 10^10^ vp HAdV5-PvDBP_RII and boosted 8 weeks later with 10 μg of PvDBP_RII protein (AP) formulated in two different adjuvants – Montanide^®^ISA720 and Abisco^®^100. Both adjuvants have previously been shown to induce high-titer responses in both AP and PPP regimes when tested with the model antigen OVA (36). For comparison, mice were also immunized three times, 21 days apart, with 10 μg PvDBP_RII protein (PPP) formulated in the same adjuvants, or with HAdV5-MVA (AM) as before. Total IgG titers were assayed in serum 2 weeks after the second protein immunization (PP) in the PPP groups, and after the final immunization in all groups by ELISA against PvDBP_RII protein (Figure 1C). Following three protein immunizations (PPP), there were no differences between the two adjuvants tested, although after two protein vaccinations (PP) Abisco^®^100 performed slightly better than Montanide^®^ISA720. Moreover, similar to previous studies using other antigens, median anti-PvDBP_RII IgG titers were over 10-fold higher in mice immunized with the AP and PPP regimes in comparison to those immunized with the AM regime [reaching significance for PPP (Abisco^®^100) versus AM following Kruskal–Wallis test with Dunn’s multiple comparison post-test, *P* < 0.01]. Overall, these data confirmed that the highest titer IgG responses against PvDBP_RII in mice can be achieved in the context of either PPP or AP immunization regimes in conjunction with relatively “strong” adjuvants.

### IgG isotype responses induced by PvDBP_RII vaccination

**Figure 2:**
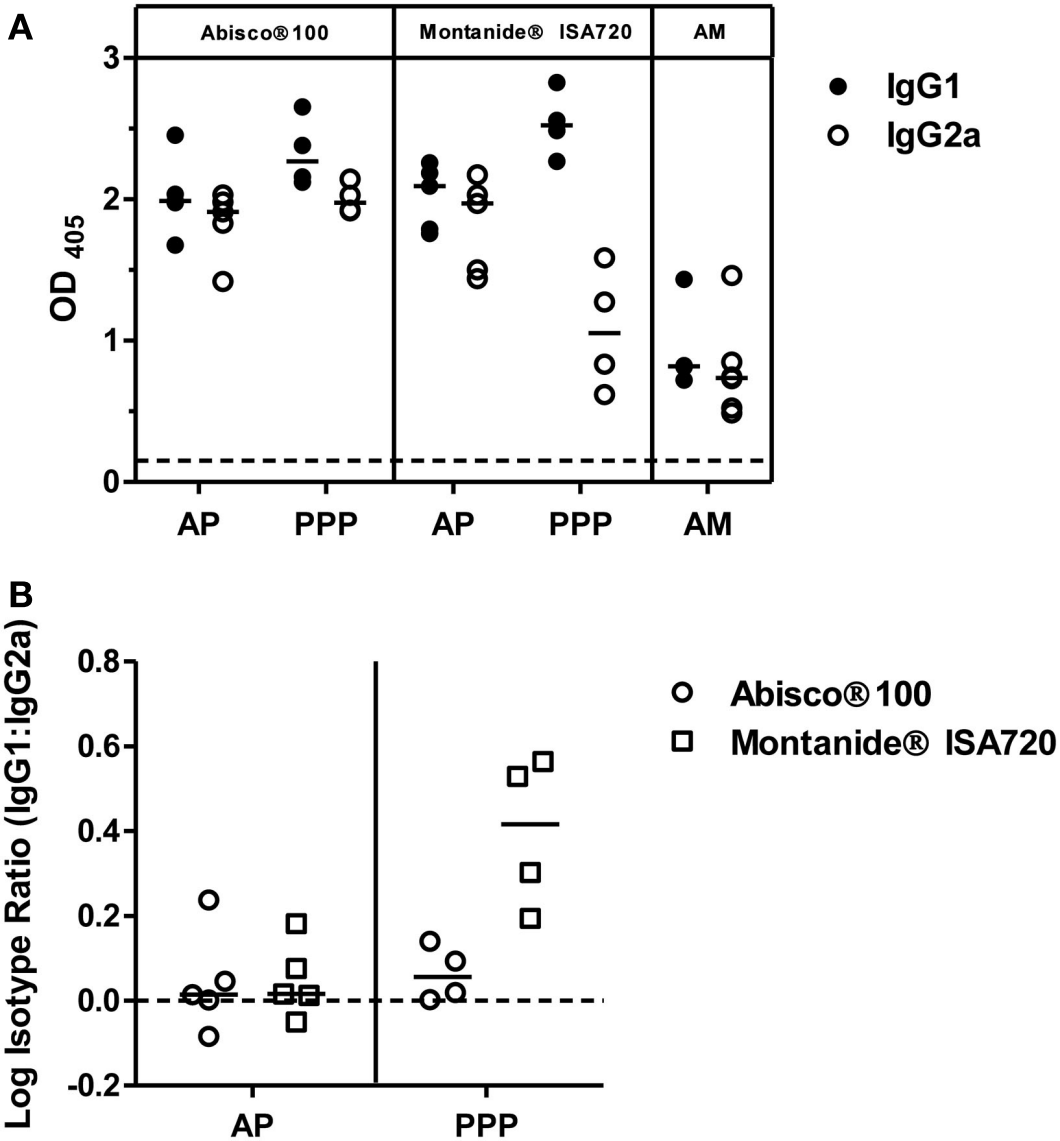
**IgG isotype responses induced by PvDBP_RII vaccination**. BALB/c mice (*n* = 4–6/group) were immunized with the AM, AP, or PPP regimes as described in Figure 1C. IgG1 and IgG2a antibody responses were measured in the serum 2 weeks after the last immunization by ELISA against PvDBP_RII protein. **(A)** Individual and median responses are shown. The dotted line indicates the threshold for responses above background determined using serum taken from the mice prior to any immunization. **(B)** IgG1:IgG2a ratios were calculated and log transformed for the AP and PPP groups. Individual and median responses are shown.

To further characterize the vaccine-induced antibody responses against PvDBP_RII, serum IgG1 and IgG2a isotype responses were assessed by ELISA 2 weeks after the final vaccination in mice immunized with the AP, PPP, and AM regimes outlined previously. Both antibody subclasses were induced by each regime tested (Figure 2A); however, as previously reported (36), a more balanced isotype response was induced in the context of AP and AM immunization – most likely reflecting stronger polarization toward Th1-type IgG2a when using a viral vector. A similar effect was observed for the Abisco^®^100 adjuvant in the context of PPP immunization, in comparison to protein formulation in Montanide^®^ISA720 which resulted in a dominant Th2-type IgG1 response. Analysis of the log isotype IgG1:IgG2a ratio showed a significant effect of immunization regime (AP versus PPP), consistent with previously published data using other antigens (36), and conferring greater induction of IgG2a following a viral vectored priming vaccination (*P* = 0.005, two way-ANOVA) (Figure 2B). The effect of adjuvant was also significant (*P* = 0.009), driven by Abisco^®^100, which induced comparable levels of IgG2a in the context of both the AP and PPP regimes.

### T cell immunogenicity of PvDBP_RII vaccines

**Figure 3:**
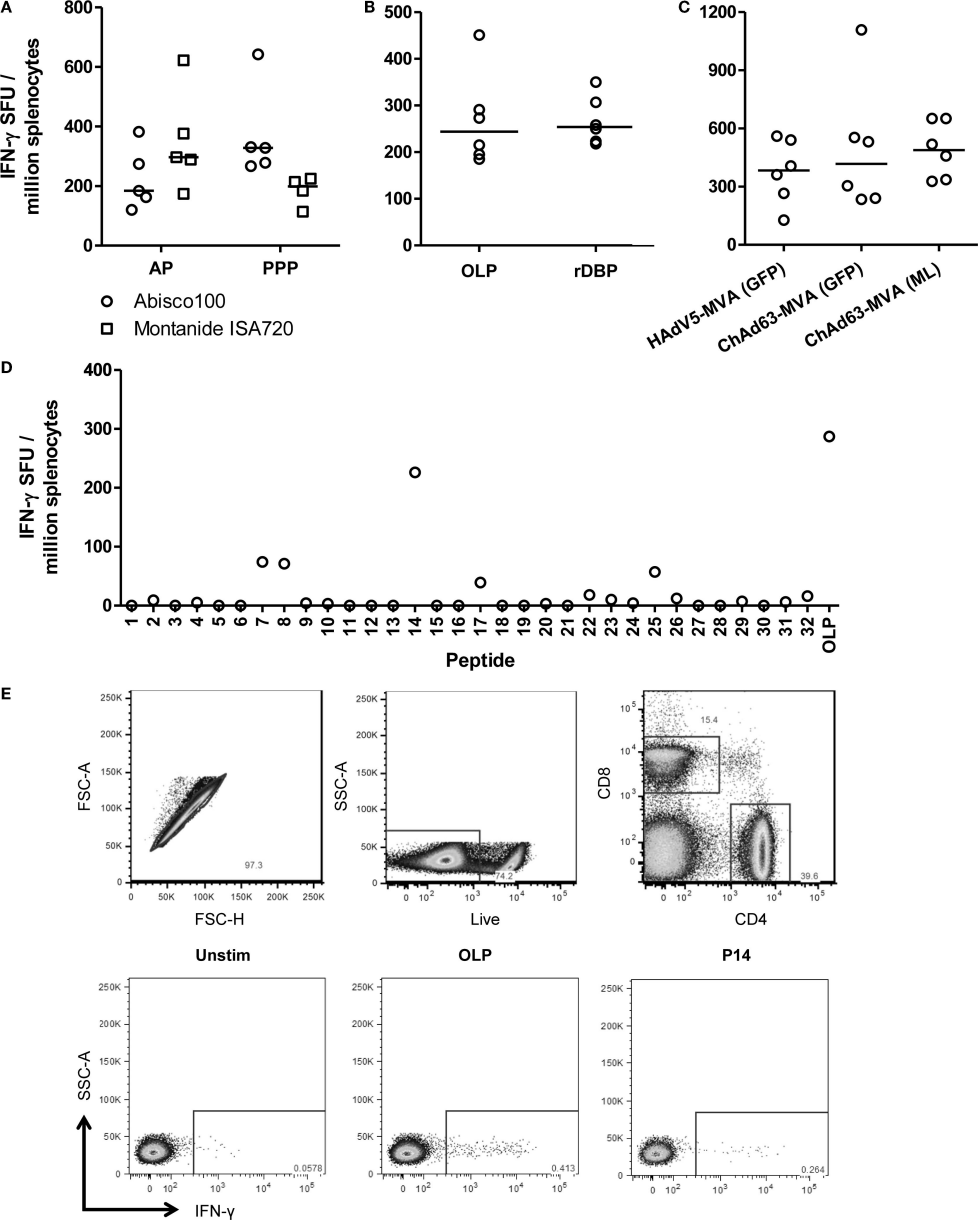
**T cell responses induced by viral vectored and protein vaccines targeting PvDBP_RII**. **(A)** BALB/c mice (*n* = 4–5/group) were immunized with AP and PPP regimes against PvDBP_RII as described in Figure 1C. Ten weeks after the last immunization, spleens were harvested and T cell responses were measured from frozen spleen samples by *ex vivo* IFN-γ ELISpot following re-stimulation with 5 μg/mL recombinant PvDBP_RII protein (rDBP). Median and individual data points are shown. **(B)** BALB/c mice (*n* = 6) were immunized with HAdV5-PvDBP_RII and boosted 8 weeks later with MVA-PvDBP_RII using doses as in Figure 1B. Two weeks after the last immunization, splenic T cell responses were measured by *ex vivo* IFN-γ ELISpot following re-stimulation with 5 μg/mL rDBP or 2 μg/mL OLP. Median and individual responses are shown. **(C)** Splenic T cell responses were measured from frozen samples in the mice reported in Figure 1B 2 weeks after the boost immunization and using OLP. Median and individual responses are shown. **(D)** BALB/c mice were immunized with 1.5 × 10^8^ ifu HAdV5-PvDBP_RII and 8 weeks later were boosted with 10^7^ pfu MVA-PvDBP_RII (GFP). Splenic T cell responses were measured from frozen spleen samples harvested 2 weeks post-boost and following re-stimulation with 5 μg/mL individual peptides (1–32) or the OLP pool control. Results from a representative mouse are shown. **(E)** BALB/c mice were immunized with 1 × 10^8^ ifu HAdV5-PvDBP_RII and 8 weeks later were boosted with 10^7^ pfu MVA-PvDBP_RII. Spleens were harvested 15 days post-boost. Intracellular cytokine staining followed by flow cytometric analysis showed the IFN-γ responses induced to the OLP pool and the three strongest peptides (Table 1) were from CD4^+^ cells. Cells were gated on lymphocytes, and then (top row) singlets by forward scatter height (FSC-H) versus area (FSC-A), then live cells (dead cell marker versus side scatter area, SSC-A), and then CD4 versus CD8. Representative plots (bottom row) of IFN-γ versus SSC-A from CD4^+^ gated cells are shown from one mouse following no stimulation (Unstim) or re-stimulation with the OLP pool or peptide 14 (P14).

The induction of CD4^+^ T cell responses is also an important consideration in the context of antibody-inducing vaccination. Such responses are necessary to help B cell responses, and drive class-switching and somatic hypermutation within the germinal centers (56). We therefore undertook an assessment of the ability of the different immunization regimes to induce PvDBP_RII-specific T cell responses. Initially, responses were assessed using an *ex vivo* IFN-γ ELISpot assay. Spleens were harvested from mice previously immunized with the AP and PPP PvDBP_RII regimes, during the resting memory phase (10 weeks after the final immunization). Splenocytes were re-stimulated for 18 h with rDBP and IFN-γ secretion assessed (Figure 3A). At this late memory time-point, moderate-to-low T cell responses were observed with a comparable magnitude irrespective of immunization regime or adjuvant. Median responses of 328 and 199 SFU/million splenocytes were induced by PPP immunization using Abisco^®^100 and Montanide^®^ISA720, respectively, while the AP regimes using the same adjuvants showed median responses of 184 and 297 SFU/million splenocytes.

In contrast to the observations here, previous studies with other antigens have generally shown stronger cellular responses when using viral vectored delivery platforms in comparison to protein-in-adjuvant (35, 36). The previous assay had also used recombinant protein for the re-stimulation phase, and not OLP which are most commonly used for such T cell assays. Protein antigen cannot bind directly to MHC molecules displayed on the surface of antigen presenting cells (APCs), and instead is most likely processed into peptides by the exogenous pathway before these are loaded onto MHC class II molecules. Re-stimulation with recombinant protein is thus unlikely to measure CD8^+^ T cell responses which require peptide presentation on MHC class I molecules. We therefore next sought to compare this assay to one using OLP, spanning the whole of the PvDBP_RII antigen. To maximize cellular responses, mice were immunized with the HAdV5–MVA regime, and spleens were harvested from mice 2 weeks after the MVA immunization. Splenocytes were subsequently re-stimulated with either OLP or rDBP in the *ex vivo* IFN-γ ELIspot assay (Figure 3B). A median response of 244 and 254 SFU/million splenocytes was observed with OLP and rDBP, respectively (*P* = 0.59, Mann–Whitney *U*-test). These data suggest that the two stimuli perform comparably, perhaps indicating minimal CD8^+^ T cell induction against this antigen in BALB/c mice. Given we previously compared antigen-specific IgG immunogenicity from the different adenoviral and MVA vaccines, we also assessed the cellular immunogenicity across the different regimes previously tested in Figure 1B. As with the humoral immunogenicity, no significant differences were observed between the groups receiving an HAdV5 versus ChAd63 prime, or the GFP marker versus “markerless” MVA boost (*P* = 0.63, Kruskal–Wallis test) (Figure 3C).

To further characterize the T cell response to PvDBP_RII, the OLP were used to map H-2^d^ T cell epitopes in these BALB/c mice. Each of the 32 individual peptides in the OLP pool were tested individually, and three peptides (7, 8, and 14) induced responses consistently above background in the tested spleen samples (Table 1 and Figure 3D). Responses were consistently strongest for peptide 14, accounting for most of the total response observed with the OLP pool. Responses against each of the three individual peptides were subsequently assessed in fresh spleen samples from HAdV5–MVA PvDBP_RII immunized mice using intracellular cytokine staining (ICS) and flow cytometric analysis. These results indicate that each of the three peptides re-stimulated CD4^+^ T cells (Figure 3E and Table 1) with no responses detected above background for CD8^+^ T cells (data not shown), in agreement with the earlier data that suggested a paucity of detectable CD8^+^ T cells responses against this antigen in BALB/c mice.

**Table 1:** **H-2^d^ T cell epitopes mapped in PvDBP_RII**.

Peptide number	Sequence
7	VNNTDTNFHRDITFRKLYLK
8	DITFRKLYLKRKLIYDAAVE
14	YSKVVENNLRSIFGTDEKAQ

*32 OLP (20mers overlapping by 10 amino acids, except the final peptide 32 which is an 18mer) were used to map T cell epitopes in BALB/c mice following HAdV5-MVA-PvDBP_RII immunization. Responses against the three peptides in the table were consistently observed by ex vivo IFN-γ ELISpot – results from a representative mouse are shown in Figure 3D. All three peptides were tested by ICS, and IFN-γ responses were observed from CD4^+^ T cells following flow cytometry (Figure 3E)*.

### Vaccine-induced antibodies inhibit the PvDBP_RII-DARC interaction

**Figure 4:**
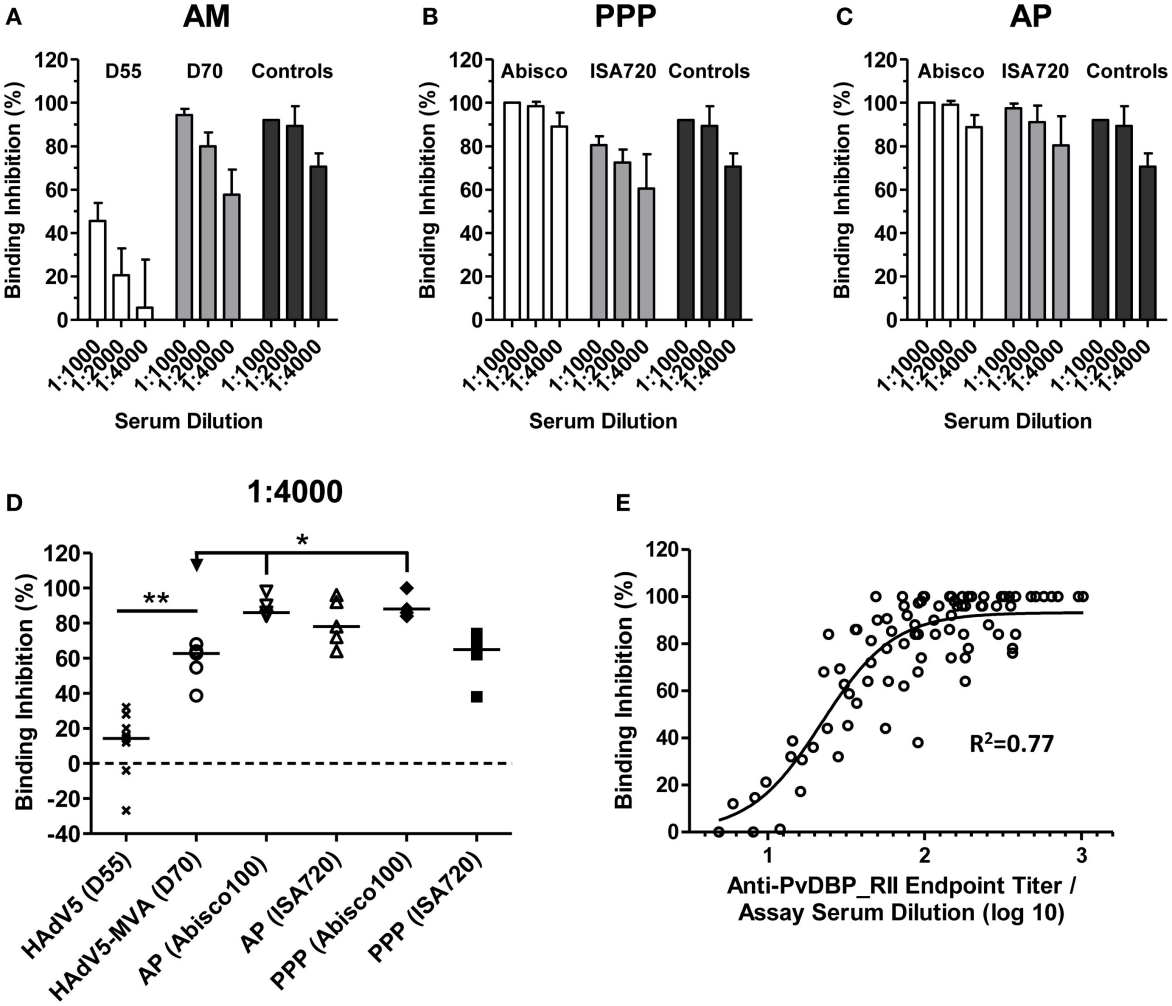
**Inhibition of PvDBP_RII binding to its receptor by vaccine-induced antibodies**. BALB/c mice (*n* = 4–5/group) were immunized with the AM, AP, and PPP regimes as described in Figure 1C. Binding inhibition assays were carried out as described in Section “Materials and Methods” using serum from the stated time-points at dilutions of 1:1000, 1:2000, and 1:4000. Each sample was run in duplicate or triplicate and the mean% binding inhibition calculated. The mean and SD of the% binding inhibition for the **(A)** AM, **(B)** PPP, and **(C)** AP groups are shown. The controls (*n* = 3) were high-titer mouse anti-PvDBP_RII serum samples. **(D)** Median and individual data points are shown for the 1:4000 serum dilution reported in **(A–C)**. Data for day 55 (D55) after HAdV5 are also included. *P < 0.05 and **P < 0.01, see Results for statistical tests. **(E)** Relationship between% binding inhibition and log_10_ anti-PvDBP_RII IgG titer assessed by endpoint ELISA. Individual data points and non-linear regression curve are shown (*n* = 87).

The rationale behind a PvDBP_RII-based vaccine relies on the induction of high-titer antibodies that inhibit RBC invasion by preventing the interaction between PvDBP and its natural ligand, the Fy antigen/DARC. In the absence of an easily available and standardized *P. vivax* assay of growth inhibition activity to assess anti-merozoite antibody function *in vitro*, a variety of *in vitro* receptor–ligand binding inhibition assays have been developed (47, 57, 58). Here, we used a version of this assay to assess the ability of vaccine-induced antibodies to inhibit binding of rDBP to the recombinant N-terminus of DARC (47). Serum samples from mice immunized with the AM, PPP, and AP regimes described above (in Figure 1C) were assayed 2 weeks after the final immunization at day 70 (D70). Sera obtained from mice at day 55 (D55) after a priming immunization with HAdV5-PvDBP_RII (i.e., prior to a MVA or protein-in-adjuvant vaccine boost) were also tested. The binding inhibition of sera was tested at three different dilutions: 1:1000, 1:2000, and 1:4000 (Figures 4A–C).

High levels of binding inhibition, >90%, were achieved with sera from mice immunized with the AM regime when tested at 1:1000, and this effect was reduced following dilution of the serum (Figure 4A). Inhibition of binding was significantly greater when compared to that after a single HAdV5 immunization, in agreement with the increase in IgG titers following the MVA boost (Figure 1A). Binding inhibition observed in the AM group was largely comparable to that achieved by PPP immunization when using the Montanide^®^ISA720 adjuvant. Encouragingly, PPP immunization with the Abisco^®^100 adjuvant, or AP immunization using either adjuvant, showed similar high-level binding inhibition at 1:1000 serum dilution, which was better maintained at 1:4000 dilutions.

Comparison across all regimes at the 1:4000 serum dilution confirmed low levels of binding inhibition (median 14.4%) in mice 55 days after HAdV5-PvDBP_RII immunization (Figure 4D). Following a boost with MVA-PvDBP_RII (GFP), binding inhibition titers were significantly increased (median 62.7%) (*P* = 0.003, Mann–Whitney test). In comparison to the PPP and AP regimes, titers achieved by the AM viral vectors were not significantly different to Montanide^®^ISA720 immunized mice (median 65.0% for PPP and 78.0% for AP). However, PPP and AP immunization using Abisco^®^100 showed a significant improvement over the AM regime (medians of 88.0 and 86.0%, respectively) (*P* < 0.05, Kruskal–Wallis test). There was also a strong sigmoidal relationship between binding inhibition and antigen-specific IgG titer, as measured by ELISA, across all tested samples and dilutions (Figure 4E), similar to other *in vitro* antibody assays measuring the functional activity of anti-merozoite antibodies (55, 59). Overall, these data indicated that the highest titers of binding inhibitory antibodies could be achieved in mice using an AP immunization regime or a PPP regime when formulating the protein vaccine in Abisco^®^100.

### Recognition of native parasite antigen by vaccine-induced antibodies

**Figure 5:**
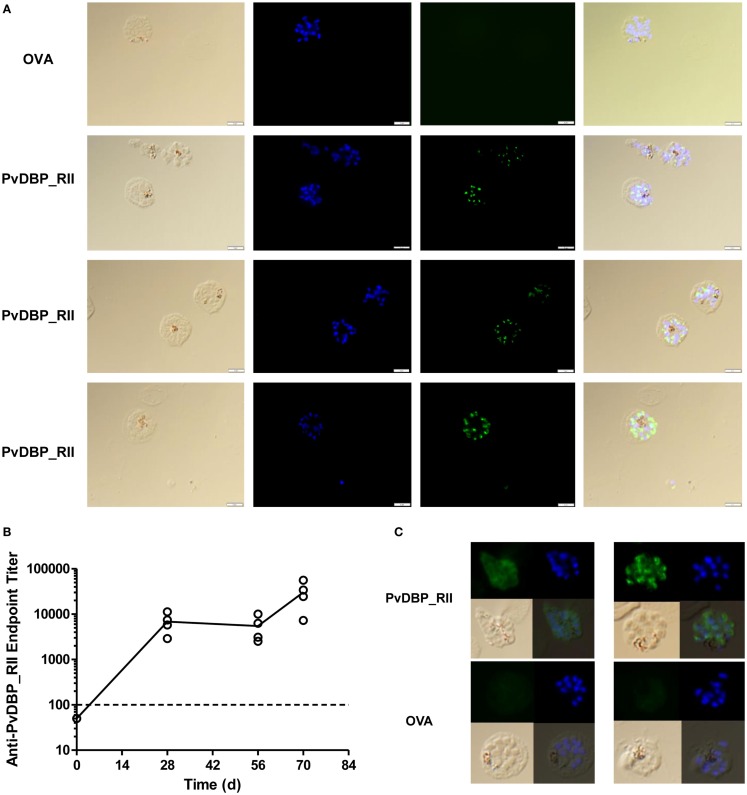
**Indirect IFA using serum from adenovirus-MVA PvDBP_RII immunized mice and rabbits**. **(A)** Indirect IFA using sera from PvDBP_RII and OVA control immunized mice (green) and microscope slides containing fixed *P. vivax-*infected cells obtained from patients in Thailand. Representative images are shown for both sets of sera. Nuclei were stained with DAPI. The merge plus bright field are also shown. **(B)** ZiKa rabbits (*n* = 4) were immunized with HAdV5-PvDBP_RII on day 0 and MVA-PvDBP_RII on day 56. Serum was harvested before immunization (day 0) and 28, 56, and 70 days post-adenovirus administration. Serum IgG titers were determined by endpoint ELISA. Individual responses are shown and the solid line indicates the mean. The dotted line indicates the cut-off for a positive response – samples tested at 1:100 dilution that did not show an OD_405_ nm reading above negative control sera are plotted below this line. Rabbits immunized with the same vectors encoding OVA showed no detectable responses in the same ELISA assay (not shown). **(C)** Indirect IFA as in **(A)** using sera from the PvDBP_RII and OVA immunized rabbits (green). Two representative images are shown for both sets of sera.

The *in vitro* binding inhibition assay utilizes rDBP, and does not confirm that vaccine-induced antibodies are capable of recognition of native antigen within the *P. vivax* parasite. In order to assess this, indirect IFAs were performed using slides prepared from the blood of *P. vivax* infected Thai patients. Slides were probed with serum taken 2 weeks after the final vaccination from mice immunized with the ChAd63-PvDBP_RII and MVA-PvDBP_RII (ML) vectors (AM regime) outlined above in Figure 1B. All of the sera tested from PvDBP_RII immunized mice were positive by IFA with a punctate staining pattern, suggesting binding of antibody to PvDBP antigen within the micronemes of daughter merozoites (Figure 5A). We also immunized rabbits with the HAdV5 and MVA vectors expressing PvDBP_RII. These rabbit sera recognized PvDBP_RII antigen by ELISA, with responses after the priming immunization showing a different kinetic to mice (Figure 5B), but similar to that seen in rabbits in previous studies with the AM regime (45, 49). Serum from these rabbits, but not from vector immunized controls, also recognized *P. vivax* schizonts with a punctate staining pattern (Figure 5C).

## Discussion

It is almost 40 years since the first report that identified the Duffy blood group antigen as a critical determinant of susceptibility to *P. vivax* malaria (15). However, despite great strides in our understanding about the nature of the interaction between the PvDBP and its receptor, it has proved challenging to translate vaccine candidates based on this paradigm into early phase proof-of-concept clinical testing. As for many malaria vaccine candidates, this can be due to difficulties with scale-up and manufacture of clinical-grade protein antigen, access to effective adjuvants or delivery formulations suitable for human use, as well as finite resources (2, 27). More recently, advances in subunit vaccine delivery platforms have made it relatively easier to raise strong immune responses against target antigens of interest in humans. One leading approach has seen the development of recombinant viral vectored technologies that allow for *in situ* expression of the encoded antigen from virally infected cells, leading to the concomitant induction of strong cellular and humoral immunity (28, 51, 54). Notably, recombinant adenovirus and poxvirus vectors are now in clinical trials ranging from Phase I–III, as vaccine candidates against numerous pathogens including *P. falciparum* malaria (33, 60), HIV-1 (61), hepatitis C virus (62), *Mycobacterium tuberculosis* (63), influenza virus (64), and Ebola (34). In this study, viral vectored vaccines, based on both human and chimpanzee adenovirus serotypes as well as the poxvirus MVA, were generated and then assessed for their ability to raise cellular and humoral immune responses against the blood-stage *P. vivax* antigen PvDBP_RII in mice and rabbits. Moreover, immune responses elicited by viral vectored vaccines encoding PvDBP_RII were compared with responses elicited by immunization with rDBP formulated with strong experimental adjuvants that have been used in human clinical trials (65, 66).

These studies confirmed that PvDBP_RII is immunogenic and elicits strong antibody responses when delivered either by adenoviral and MVA vectors or as recombinant protein formulated with adjuvant. Anti-PvDBP_RII-specific IgG antibody responses were induced by viral vectored vaccines, and these followed a similar kinetic to that previously observed in mice and rabbits in the context of a standard heterologous prime-boost (AM) regime with other candidate antigens (29, 36, 45, 49, 50). Consistent with studies using other malaria transgenes (45, 49, 50), there was no significant difference between IgG titers 2 weeks after a priming immunization with either HAdV5 or ChAd63, although there was a trend toward weaker antibody titers with ChAd63. Following a boost with recombinant MVA, and consistent with numerous other studies (45, 49, 67, 68), anti-PvDBP_RII titers and IFN-γ T cell responses were comparable irrespective of priming adenovirus vector, or use of a GFP marker gene in the recombinant MVA. In recent years, ChAd63 and markerless MVA vectors encoding target antigens from *P. falciparum* have been routinely progressed to Phase I/II clinical trials (44, 53, 69, 70), and the data here suggest the vectors for PvDBP_RII are equally suitable for clinical development.

Protection against blood-stage merozoites requires high concentration of functional antibody, capable of inhibiting the rapid RBC invasion process (32, 71). More recently, “mixed modality” adenoviral prime – protein-in-adjuvant boost regimes have been developed, and in a recent Phase Ia clinical trial with PfAMA1 antigen, were shown to induce higher antigen-specific IgG responses than the standard AM regime (55). Given the availability of a protein PvDBP_RII vaccine that is also being progressed to clinical development (72), we also explored the utility of the AP mixed-modality approach with this antigen and two “strong” adjuvants that have been in clinical testing (65, 66). At the doses tested, the AP regime, using both Montanide^®^ISA720 and the Abisco^®^100 adjuvants, significantly outperformed the AM regime. In comparison to protein-only regimes, the AP regime performed comparably to two protein immunizations (PP) with Abisco^®^100, but tended to improve upon Montanide^®^ISA720. Following three protein immunizations (PPP), all regimes performed comparably in terms of the total anti-PvDBP_RII IgG response. These regimes also elicited comparable levels of IFN-γ T cell responses as measured by ELISpot assay, and in the case of BALB/c mice, these appeared largely composed of a CD4^+^ T cell response directed against one dominant, and at least two sub-dominant, epitopes. Natural exposure to *P. vivax* (73), as well as immunization of rhesus macaques with PvDBP_RII protein in Montanide^®^ISA720 (24), also elicits antigen-specific T cell responses detectable by ELISpot. Their role in providing help to B cell responses and/or contribution to acquired immunity remains to be better defined.

Consistent with previous reports (35, 36), adenoviral priming also skewed the antibody response toward the Th1-type IgG2a cytophilic isotype, especially in the case of Montanide^®^ISA720. The potential role of the equivalent human isotypes IgG1 and IgG3 in natural immunity against *P. vivax* remains unclear, although these subclasses tend to dominate in the natural response to merozoite antigens for both *P. vivax* and *P. falciparum* (74, 75) and likely mediate effector functions via their Fc including interactions with immune cells (76–78) and complement (79). The ability to tailor the quality of the antibody response by using different vaccination regimes in humans may allow for this question to be better addressed in the future. In a similar vein, the ability of different protein vaccine adjuvants to affect the “quality” or fine-specificity of the vaccine-induced IgG has been reported in some cases (80), but not others (81), and will warrant further investigation in the context of clinical trials.

The ability of vaccine-induced antibodies to block binding of PvDBP_RII protein to its receptor DARC was assessed here using an *in vitro* ELISA-based binding inhibition assay against the vaccine homologous *Sal*I allele. The highest levels of binding inhibition were achieved with sera from mice immunized using the AP and PPP regimes containing Abisco^®^100 at a dilution of 1:4000, with comparable but slightly lower inhibition seen at the same dilution when using sera from mice immunized with the AM regime or protein in Montanide^®^ISA720. These levels were comparable, if not better, than those previously reported in similar mouse studies (42). Notably, binding inhibition also correlated with IgG titers, as previously reported for this assay using sera from immunized rhesus macaques (24).

The antibodies induced here in mice and rabbits by the AM regime were also capable of recognizing native *P. vivax* parasite antigen as assessed by IFA using freshly isolated parasites from Thai patients, consistent with other studies using viral vectors to deliver leading *P. falciparum* antigens in rabbits and humans (31, 44). It is encouraging that these antibodies can recognize native parasite isolates. Studies of naturally acquired immunity following *P. vivax* exposure have reported the induction of strain-specific immunity (82, 83), and numerous sequence polymorphisms have been found within the PvDBP_RII antigen with the majority localized to subdomain 2 (SD2) (84, 85). Other studies have suggested that the more highly conserved SD3 is also important for PvDBP_RII engagement with DARC and can elicit strain-transcending blocking inhibitory antibodies (86). Indeed, the development of naturally acquired cross-reactive anti-PvDBP_RII antibodies that recognize and block binding of diverse PvDBP_RII domains from *P. vivax* field isolates have been reported albeit at low frequency (26). This may be due to the limited exposure time of the PvDBP_RII micronemal ligand prior to binding the DARC receptor.

In contrast to epidemiological data, preclinical immunogenicity studies with the *Sal*I allele of PvDBP_RII have shown that this immunogen is capable of eliciting high-titer, cross-reactive binding-inhibitory antibodies in the ELISA-based assay (80, 86) and inhibitory capacity using an *ex vivo* invasion assay (87). These data suggest that PvDBP_RII vaccination using either viral vectors or recombinant protein formulated with adjuvant could elicit antibodies that quantitatively and qualitatively differ from those induced by natural exposure. Whether this translates into the development of potent immune responses in humans that cross-react with diverse variants, and provide protection against *P. vivax* blood-stage infections, will be important future questions for clinical trials with first-generation vaccines based on PvDBP_RII.

Overall, these data confirm the immunogenicity of clinically relevant viral vectors in small animal models. The ChAd63 and MVA vectors encoding PvDBP_RII (*Sal*I) have since progressed to Phase Ia clinical testing in healthy adults in Oxford, UK (Clinicaltrials.gov NCT01816113). Given the on-going development of a PvDBP_RII protein-based vaccine toward clinical manufacture (72), it will also be possible to test mixed-modality regimes in Phase Ia trials in due course, as recently performed for the PfAMA1 antigen using ChAd63, MVA, and protein-in-adjuvant (55). The clinical data with ChAd63 and MVA will provide the first opportunity to assess the safety and immunogenicity of the PvDBP_RII antigen in humans, and enable detailed insight into the human vaccine-induced antibody response against this leading target antigen.

## Author Contributions

Conceived, designed, and performed the experiments: SC, RS, DL, SE, JC, AG, JJ, AD, RS, CC, SD. Analyzed the data: SC, RS, CC, SD. Contributed reagents/materials/analysis tools: FN, LR, BR, CC. Wrote the paper: SC, SD.

## Conflict of Interest Statement

Simone C. de Cassan, Anna L. Goodman, Alexander D. Douglas, and Simon J. Draper are named inventors on patent applications covering malaria vectored vaccines and immunization regimes. Chetan E. Chitnis is a named inventor on a patent covering PvDBP_RII. The remaining authors have no conflict of interest to declare.
